# Dehydroepiandrosterone (DHEA) supplementation in diminished ovarian reserve (DOR)

**DOI:** 10.1186/1477-7827-9-67

**Published:** 2011-05-17

**Authors:** Norbert Gleicher, David H Barad

**Affiliations:** 1 Center for Human Reproduction (CHR) and Foundation for Reproductive Medicine, New York, NY, USA; 2 Department of Obstetrics, Gynecology and Reproductive Sciences, Yale University School of Medicine, New Haven, CT, USA; 3 Departments of Epidemiology and Social Medicine and Obstetrics, Gynecology and Women's Health, Albert Einstein College of Medicine, Bronx, NY, USA

## Abstract

**Background:**

With infertility populations in the developed world rapidly aging, treatment of diminished ovarian reserve (DOR) assumes increasing clinical importance. Dehydroepiandrosterone (DHEA) has been reported to improve pregnancy chances with DOR, and is now utilized by approximately one third of all IVF centers world-wide. Increasing DHEA utilization and publication of a first prospectively randomized trial now warrants a systematic review.

**Methods:**

PubMed, Cochrane and Ovid Medline were searched between 1995 and 2010 under the following strategy: [<dehydroepiandrosterone or DHEA or androgens or testosterone > and <ovarian reserve or diminished ovarian reserve or ovarian function >]. Bibliographies of relevant publications were further explored for additional relevant citations. Since only one randomized study has been published, publications, independent of evidence levels and quality assessment, were reviewed.

**Results:**

Current best available evidence suggests that DHEA improves ovarian function, increases pregnancy chances and, by reducing aneuploidy, lowers miscarriage rates. DHEA over time also appears to objectively improve ovarian reserve. Recent animal data support androgens in promoting preantral follicle growth and reduction in follicle atresia.

**Discussion:**

Improvement of oocyte/embryo quality with DHEA supplementation potentially suggests a new concept of ovarian aging, where ovarian environments, but not oocytes themselves, age. DHEA may, thus, represent a first agent beneficially affecting aging ovarian environments. Others can be expected to follow.

## Background

Casson and associates were first to suggest therapeutic benefits from supplementation with dehydroepiandrosterone (DHEA) in women with diminished ovarian reserve (DOR) [1]. They also suggested that, in micronized form, the androgen offers potential for postmenopausal steroid replacement, adjunctive to estrogen [2]; that its conversion may not be symmetrical, favoring androgens over estrogen, with testosterone increasing and estradiol remaining low [2]; that DHEA has immunomodulatory effects [3], now therapeutically explored in autoimmune diseases [4,5], that vaginally administered DHEA, while delivering equivalent hormone, substantially diminishes bioconversion comparatively to oral micronized products [6], and that abnormally low adrenal DHEA secretion is potentiated by ovarian hypertstimulation with gonadotropins [7].

They also reported that DHEA is well tolerated and increases IGF-1 levels [8]. A main focus of this group's work was, thus, the compensation of adrenal cortical changes in aging women with DHEA [9].

Their initial therapeutic use of DHEA in patients with DOR [1] was motivated by observed increases in IGF-1 after DHEA supplementation [8]. Since growth hormone had been suggested to improve oocytes yields via IGF-1, they hypothesized that DHEA may be able to achieve similar effects. Though demonstrating improvement in oocytes yields [1], their initial paper went unnoticed for years, and initiated no follow up studies.

It was left to a 43 year old infertility patient to rediscover their paper, searching the literature for remedies to overcome DOR. She, in a first in vitro fertilization (IVF) cycle, had produced only a single egg and embryo, and was advised to consider oocyte donation [10]. This lay-person, reviewing the medical literature, amongst various suggested treatment options for improving low egg counts, chose DHEA because it was the only medication in the United States (US) available without prescription (DHEA in the U.S. is considered a food supplement).

**Figure 1:**
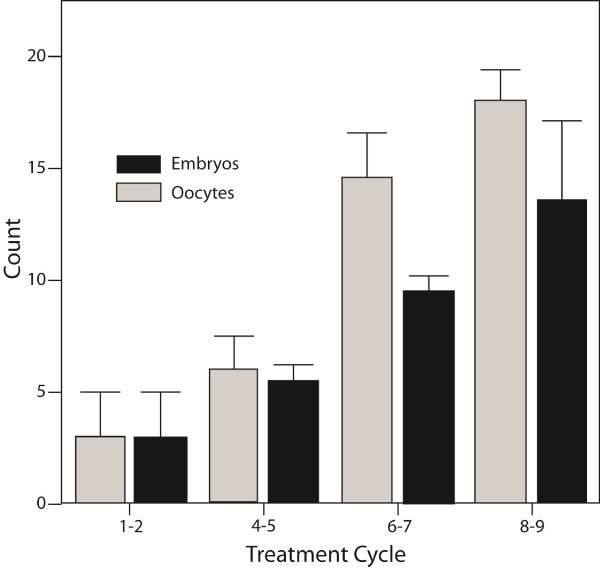
**Oocyte and embryo counts in index patient**. The patient underwent nine consecutive IVF cycles and increased oocytes and embryo yields from cycle to cycle, starting with one egg and embryo, respectively, and ending up with 17 oocytes and 16 embryos in her ninth cycle. Gonadotropin stimulation was reduced in her last cycle for concerns about possible ovarian hyperstimulation. The patients advised us of her DHEA supplementation only after her sixth cycle. The figure is modified from Barad and Gleicher, with permission, [10].

In a second IVF cycle she produced three oocytes/three embryos. Her oocyte and embryo yields after that increased from cycle to cycle (Figure 1). In the ninth IVF cycle, now age 44, gonadotropin dosages had to be reduced because of concerns about potential ovarian hyperstimulation, she still produced 17 oocytes (16 embryos) in that cycle alone.

Following nine consecutive all-freeze IVF cycles, her change in ovarian function under DHEA supplementation (unknown to her physicians until after her 6th cycle) initiated the prospective investigation of DHEA [10]. Above noted initial patient will here be referred to as "index patient." Six years following this patient's self-administration of DHEA, a recent survey of IVF centers concluded that approximately one third of all IVF centers world-wide have started DHEA supplementation in women with DOR [11].

Because patients, largely, were not willing to enter randomization, a clinical trial of DHEA in the US (ClinicalTrials.gov ID# NCT00419913) had to be abandoned. Considering the usually limited time for conception left for DOR patients, this cannot surprise. A multicenter European trial involving centers in Austria, Switzerland and the Czech Republic, designed in follow up to the cancelled U.S. trial, had to be abandoned for the same reasons. Only recently did an Israeli group, for the first time, succeed in completing a small, prospectively randomized study [12]. All other DHEA studies published so far relied on other study designs.

An excellent recent study in a mouse model also offers considerable support for DHEA supplementation. This study very convincingly demonstrates the critical importance of androgens in regulating ovarian development and function [13]. In very elegantly designed experiments, Sen and Hammer demonstrated that androgens promote preantral follicle growth, while preventing follicular atresia. Androgens, long considered antagonistic to normal follicle development, thus, suddenly assume a central role in follicular development and female fertility [13]. Noting the previously referred to preferential conversion of DHEA to testosterone [2], these observations offer a potential mechanism by which DHEA supplementation improves ovarian function.

## Methods

As only one, small prospectively randomized study, addressing DHEA supplementation with DOR, has so far been published (12), this review presents a comprehensive summary of *all *published data, indiscriminate of study format and/or quality assessment. Limitations of presented data are, however, discussed in detail.

### Search strategy, study selection, data collection and analysis

We searched PubMed, Cochrane and Ovid Medline between 1995 and 2010 for all publications under the following key words: Dehydroepiandrosterone or DHEA; androgens or testosterone; ovarian reserve or diminished ovarian reserve; ovarian function or diminished ovarian function. In addition, we explored the bibliographies of all relevant publications for further relevant citations, which had not been detected via the original search. So identified publications were also in detail reviewed by the authors, including their relevant citations. A total of 114 publications were, thus, reviewed for this publication, with 64 being cited in this manuscript. The 50 manuscripts reviewed but not referenced in the review either contained no relevant information in regards to the topic of this review and/or only recited data of earlier published manuscripts, which are included in the reference list of this manuscript.

Every published study, addressing DHEA supplementation in infertile women with DOR, was reviewed and is cited in this manuscript. No selection of materials for inclusion or exclusion, therefore, took place. All publications were reviewed by both authors, who agreed with analysis and interpretation of data.

### IRB approval

Since here reviewed data were based on prior publications, no Institutional Review Board (IRB) approval was required for this study. All materials from the authors own center had previously been accumulated (and published) after appropriate IRB review.

## Results

### Clinical experience

#### Increase in oocytes and embryo yields

Casson et al. did not claim direct DHEA effects on DOR ovaries. They, instead, suggested that DHEA supplementation appears to augment ovarian stimulation with gonadotropins in poor responders, resulting in improved oocytes yields [1]. Likely due to their small study population, their paper failed to elicit follow up until previously noted index patient, five years later, rediscovered their publication [10].

Like the paper by Casson et al [1], the index patient's experience initially suggested that improvements from DHEA supplementation were primarily quantitative (better oocyte yields), and even greater than originally reported by the Baylor group. Moreover, the index patient's longitudinal experience over nine IVF cycles also demonstrated continuous improvements in oocyte (and embryo) numbers with increasing length of DHEA supplementation.

Cumulative DHEA effects over time, in turn, suggested possible effects on follicle recruitment or, as previously reported by the Baylor group, a synergistic effect between DHEA and gonadotropins [8]. One, therefore, at that point could conclude that DHEAsupplementation, potentially, may not only offer improving oocyte numbers, but also improving ovarian reserve (OR). Since the Baylor group had only attempted to address the problem of poor response to ovarian stimulation [1], this conclusion represented a significant expansion of the concept underlying the utilization of DHEA.

Concentrating on DHEA effects on OR significantly changed concepts since OR, defined by size and quality of remaining follicles within ovaries [14,15], presumed DHEA effects on ovaries beyond just one stimulation cycle. DHEA would then not only have to impact oocyte and embryo numbers but also oocyte and embryo quality and, therefore, ultimately, pregnancy success. In absence of prospectively randomized studies, and with use of other study formats, conclusions, of course, have to be drawn cautiously.

#### Improvements in oocytes and embryo quality

The first 25 DOR patients, supplemented with DHEA in paired analysis of pre- and post-DHEA cycles, confirmed significant increases in oocytes and embryo numbers, previously observed in the index patient [16]. They, however, also demonstrated improved embryo quality, including better embryo grades, average embryo scores and, most importantly, better embryo numbers available for transfer.

Since low embryo numbers are a principle characteristic of DOR, this observation further supported the hypothesis that DHEA may also positively affect pregnancy chances. Uniformity of quantitative and qualitative IVF outcome improvements (Table 1) also encouraged such thought.

**Table 1:** Comparisons of pre- and post-DHEA cycles in 25 women with DOR*

	Pre-DHEA	Post-DHEA	p-value
Cycle cancellations (%)	32.0	4.3	0.02
Number oocytes	3.4 ± 0.5	4.4 ± 0.5	<0.05
Fertilized oocytes (n)	1.4 ± 0.3	3.0 ± 0.5	<0.001
(%)	39	67	<0.001
Day 3 blastomeres	3.4 ± 0.4	4.7 ± 0.5	0.01
embryo grade	2.9 ± 0.1	3.4 ± 0.1	0.02
Cumulative embryoscore/oocytes	8.4 ± 1.5	16.1 ± 1.6	0.001
Number of transferred embryos	1.4 ± 0.2	2.4 ± 0.3	0.005
Normal day 3 embryos	1.2 ± 0.2	2.7 ± 0.4	0.001

* 25 patients were evaluated in their respective IVF cycle outcomes pre- and post-DHEA. This study design potentially biases outcome against positive DHEA effects since patients who entered DHEA supplementation after a prior failed IVF cycle, quite obviously, reflected, in view of their prior IVF treatment failure, a negatively selected patient population. Pre- and post DHEA cycles occurred at ages 39 ± 0.8 and 40.4 ± 0.8 years, respectively, also mildly biasing the study against positive DHEA findings. Post-DHEA patients were on supplementation 17.6 ± 2.13 weeks by time of second IVF cycle. Data extracted from Barad and Gleicher [16].

#### Improvements in pregnancy rates

In a subsequently larger cohort of 89 DOR patients, supplemented with DHEA for up to four months, and in 101 controls, DHEA patients demonstrated shorter time to pregnancy and higher pregnancy rates (cumulative clinical pregnancies, 28.1% vs. 10.9%; 95% CI 1.2-11.8; p < 0.05), despite prognostically more favorable controls (more oocytes, P < 0.01; normal day-3 embryos, P < 0.05; and more embryos transferred, P < 0.05). Moreover, study patients were also older (41.6 ± 0.4 vs. 40.0 ± 0.4 years) [17].

DHEA thus improved all outcome parameters, even though patient selection was biased against such findings. This study for the first time also suggested primacy of egg and embryo quality over egg and embryo quantity.

[Though not part of here reported literature review, we find it noteworthy that, concomitantly, Edward Ryan's Toronto West Fertility Center had started utilizing DHEA in women with DOR. This group in subsequent years in a number of abstracts reported significantly improved clinical pregnancy rates in hundreds of IVF and insemination cycles, using varying ovarian stimulation protocols (Ryan E, Personal communication, 2009). In cooperation with Robert F Casper from Toronto's Mount Sinai Hospital and University of Toronto, they more recently reported on 47 patients with prior clomiphene citrate failures who, supplemented with 75 mg DHEA daily for at least 60 days prior to inseminations, with stimulation by either clomiphene citrate or letrozole in combination with FSH. Controls were 46 women, matched by age and baseline FSH, without supplementation. DHEA patients demonstrated significantly higher antral follicle counts, significantly improved pregnancy rates (29.8 vs. 8.7%; CI 1.3-14.8) and live births (21.3% and 6.5%, respectively) [18], numbers remarkably similar to those earlier reported from our center [17].]

From Turkey, Sönmezer and associates reported on 19 "poor responders" to ovarian stimulation [19]. After DHEA, this group experienced significant decreases in cycle day-3 estradiol levels, increased large follicle numbers, MII oocytes, top quality day-2 and day-3 embryos, reduced cycle cancellations and improved pregnancy rates per patients (47.4% vs. 10.5%, P < 0.001) and per embryo transfer (44.4% vs. 0.0%, P < 0.01).

Wiser and associates most recently presented the first prospectively randomized study of DHEA supplementation with DOR (ClinicalTrial.gov ID # NCT01145144) (12). While small (17 study and 16 control patients), DHEA patients demonstrated improved embryo quality over time (P = 0.04), with increasing length of DHEA supplementation and significantly higher live birth rates (23.1 vs. 4.0%; P = 0.05).

While in our opinion the study was underpowered since the authors counted 55 IVF cycles in 33 patients, thus including repeat IVF cycles without evidence of adjustments via the randomization schedule, it, nevertheless, has to be considered a milestone in view of prior failed attempts to conduct such studies.

#### Premature versus physiologic DOR

**Figure 2:**
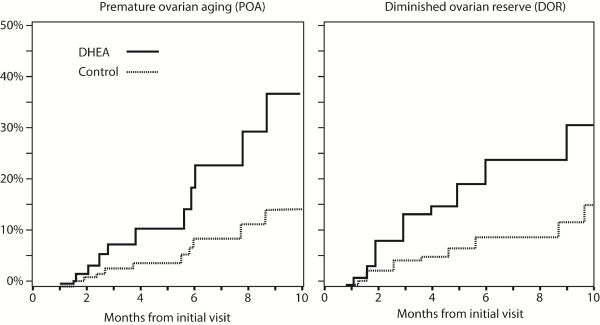
**Cumulative pregnancy rates in women with DOR with and without DHEA supplementation**. The figure demonstrates on the left side cumulative pregnancy rates in DHEA and control patients with POA (for definition see text). The right side of the figure demonstrates cumulative pregnancy rates in women above age 40 years. Both patient populations demonstrate similar treatment benefits for DHEA, though POA patients appear to have a slight pregnancy advantage, further confirmed in later data presentations. Modified with permission from Barad et al [17].

When DOR patients were separated into those with age-dependent DOR and women with so-called premature ovarian aging (POA) [17], also given the acronym occult primary ovarian insufficiency (OPOI) (20), DHEA supplementation proved similarly effective in both groups, though POA patients did mildly better. The beneficial effects of DHEA increased with length of DHEA supplementation, documented by increasing discrepancy in cumulative pregnancy rates between the groups over time (Figure 2) [17].

This confirmed initial observations in the index patient [10]. DHEA effects occur relatively quickly (apparently within ca. 2 months) but peak only after 4-5 months of DHEA supplementation. Our center, therefore, supplements DHEA for at least six weeks prior to IVF cycle starts, though even longer pretreatment may be used in younger patient.

Surprising numbers of spontaneously conceived pregnancies during these waiting periods suggest that DHEA, alone, can in DOR patients raise fecundity [17].

#### Premature ovarian failure (POF)/primary ovarian insufficiency (POI)

POA/OPOI has to be differentiated from outright premature ovarian failure (POF), also called primary ovarian insufficiency (POI) [20]. Mamas and Mamas claimed a small case series of five alleged POF/POI patients, who spontaneously conceived while on DHEA [21].

Intriguing in concept, the report should, however, be viewed with caution since three of the five reported patients do not qualify for a diagnosis of POF/POI and, likely, more resemble POA/OPOI patients [22]. Mamas and Mamas, however, reiterated their claim [23] and in a personal communication advised us of additional pregnancies in DHEA supplemented POF/POI patients (Mamas L, Personal communication, ESHRE Annual Meeting, Amsterdam, The Netherlands, July 2009).

Anecdotally, we recently recorded, after 4 months of DHEA supplementation, a spontaneous pregnancy in a 38 year old woman with POF/POI (highest recorded FSH 100.0 mIU/mL). A registered clinical trial of DHEA in POF/POI patients (ClinicalTrials.gov ID#NCT00948857) is currently underway at our center but is not expected to yield results for at least two more years.

#### Effects on embryo ploidy, miscarriage risk and live birth rates

In her last IVF cycle the index patient offered to have 10 of her embryos investigated for aneuploidy [10].

Only one was euploid. Limitations in current methods of preimplantation genetic screening (PGS) restrict applications of PGS in women with DOR since small embryo numbers mostly preclude PGS [24]. In 2007, a small pilot study demonstrated in 100 percent of DHEA treated but only 53 percent of control IVF cycles at least one euploid embryo (p < 0.05) [25]. Patient selection was again biased against DHEA since DHEA supplemented women were older than controls and, therefore, should have demonstrated higher aneuploidy rates.

Though offering statistically significant results, study results of this pilot also had to be viewed cautiously because of small study numbers and potential biases and patient selection. Better numbers and superior selection of controls in a more recently published study permitted for more reliable results, which confirmed significant decreases in aneuploidy after DHEA supplementation [26].

In absence of adequate PGS numbers, close statistical associations between aneuploidy and spontaneous pregnancy loss [27] offered an indirect way to investigate the issue. In a combined effort with Edward Ryan's Toronto center, enough DHEA pregnancies had been established to allow for a statistically robust analysis of miscarriage rates. As at least 60 percent of miscarriages are associated with chromosomal abnormalities [27], a DHEA effect on ploidy should be statistically reflected in lower miscarriage rates, and this was, indeed, confirmed [28].

Depending on statistical method utilized, pregnancy loss after DHEA supplementation was reduced by 50 to 80 percent in comparison to national U.S. IVF pregnancy rates, a conclusion further strengthened by the following: (i) Miscarriage rates in Toronto and New York were practically identical (15.2 and 15.0%, respectively); (ii) The U.S. national IVF registry, used as control population, in contrast to DHEA patients, included only relatively few DOR patients. Women with DOR are known to demonstrate significantly increased miscarriage rates in comparison to other infertility etiologies [29].

**Figure 3:**
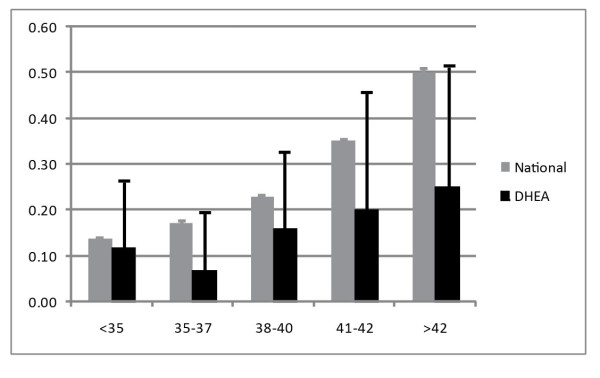
**Age-stratified miscarriage rates in DHEA supplemented DOR patient in comparison to national U.S. IVF pregnancies**. DHEA pretreated patients demonstrated significantly lower miscarriage rates at all ages. The difference was, however, relatively small under age 35 years and progressively increased after that age. Modified with permission from Gleicher et al [28].

Makeup of controls, therefore, biased the study against findings favoring DHEA supplementation; (iii) The combined miscarriage rate of 15.1 percent in DHEA pretreated patients at both IVF centers is reflective of spontaneous miscarriage rates for normal, fertile populations [30]; (iv) DHEA effects on miscarriage rates were small under age 35 years but increased progressively after that age (Figure 3).

Increasing aneuploidy with advancing female age, of course, would suggest increasing effectiveness of DHEA with advancing female age. A beneficial DHEA effect on embryo ploidy, therefore, appears likely, and seems to increase with age.

**Figure 4:**
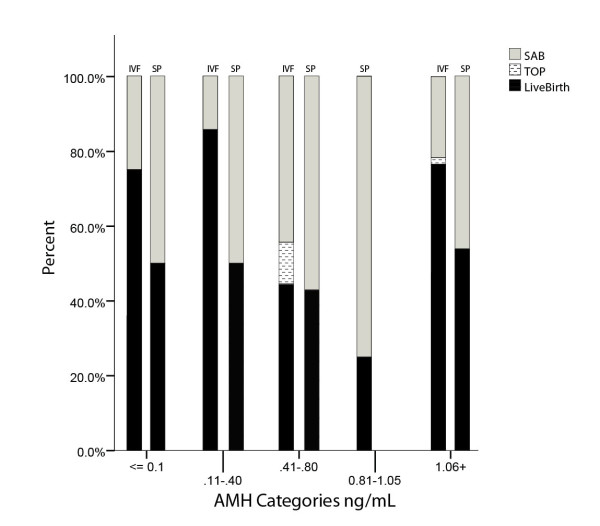
**Spontaneous pregnancy loss in spontaneous and IVF pregnancies at various AMH levels**. The figure depicts at various AMH levels in the left column IVF pregnancies (IVF), as previously reported [Gleicher *et al*. (31)], and in the right column spontaneously conceived pregnancies (SP). Each column represents 100% of all pregnancies established, separated for live births (black section), voluntary termination of pregnancy (TOP; usually for aneuploidy) and spontaneous miscarriages (SAB). The figure demonstrates that at very low AMH levels (≤0.40 ng/mL) and at AMH ≥ 1.06 ng/mL. IVF pregnancies led to significantly higher live birth rates than spontaneously conceived DHEA pregnancies. Lowest pregnancy and live birth rates were observed with IVF and spontaneously between AMH 0.41-1.05 ng/mL, with no spontaneous DHEA pregnancies at all at AMH 0.81-1.05 ng/mL. While in IVF pregnancies miscarriage rates were clearly reduced at very low and at higher AMH, miscarriages appeared unaffected (~50%) in spontaneously conceived pregnancies.

Recent data further support these conclusions (Figure 4): Miscarriage rates, even with most severe DOR, are very low after DHEA supplementation. Between non-detectable anti-Müllerian hormone (AMH) of <0.1 and 0.4 ng/mL they remain equal to those seen in normally fertile women, increase at AMH 0.41 - 1.05 ng/mL to over 50 percent of all pregnancies established, representing the expected rate in DOR patients [29], only to fall off again above AMH 1.05 ng/mL [31]. With miscarriage rates, likely due to DHEA supplementation, being very low under AMH 0.4 and above 1.05 ng/mL, the question arises why this effect is not also seen at AMH levels of 0.41-1.05 ng/mL?

Trying to find an answer, we investigated 39 spontaneous pregnancies in DOR patients on DHEA, conceived before they reached their first IVF cycle. Figure 4 depicts miscarriage rates of spontaneous in comparison to IVF pregnancies, demonstrating that spontaneous pregnancies experienced at all low AMH levels almost identically high miscarriage rates around approximately 50 percent of all pregnancies established. They, thus, demonstrate expected pregnancy loss rates for DOR patients [29], and do not seem to benefit from DHEA supplementation like IVF pregnancies.

Spontaneous pregnancies were, of course, conceived after shorter exposure to DHEA than IVF pregnancies since, as noted above, our center's DOR patients are at least for six weeks on DHEA supplementation before an IVF cycle is initiated. Patients who conceive spontaneously on DHEA during this "waiting period," therefore, by definition, had shorter DHEA exposure times. This observation then leads to the conclusion that shorter exposure times may be enough to raise fecundity but may not suffice to positively affect ploidy and miscarriage rates.

#### Predicting the effectiveness of DHEA

AMH levels are predictable of treatment outcomes after DHEA utilization [31,32]. Table 2 summarizes how AMH levels relate to chance of conception and live births in IVF pregnancies: Even with complete absence of detectable AMH, an approximately five percent pregnancy chance per IVF cycle can be obtained. Since miscarriage rates are very low, pregnancy and live birth rates are very close. Outcomes remain the same up to AMH 0.4 ng/mL, when clinical pregnancy chances approximately double. Live birth rates remain, however, unchanged since at AMH 0.41-1.05 ng/mL spontaneous pregnancy wastage increases. Above those AMH levels pregnancy chances greatly improve and miscarriage risk recedes once again to much lower levels [31].

**Table 2:** Effectiveness of DHEA supplementation in IVF pregnancies based on preconception AMH levels

DHEA effects	Reference
Pregnancies/live births at all AMH levels; Not even undetectable levels of AMH, therefore, preclude pregnancies/live births;	[31]
Pregnancies lowest ^a ^at AMH levels <0.1 (undetectable) -0.4 ng/mL, intermediate ^b ^at AMH 0.41-1.05 ng/ML and high ^c ^≥ AMH 1.06 ng/mL;	[31]
Spontaneous miscarriage rates lowest ^d ^at AMH ≤ 0.4 ng/mL and 1.06 ng/mL; Highest ^e ^at AMH 0.41-1.05 ng/mL;	[31]
Live births rates uniformly low ^f ^at AMH <0.1-1.05 ng/mL and high ^g ^at AMH ≥ 1.6 ng/mL;	[31]
AMH increases in parallel with length of DHEA supplementation;	[32]
This increase is more pronounced in younger POA than older DOR patients;	[32]
Improvement in AMH levels with DHEA supplementation is highly predictive of pregnancy success	[32]

^a^Approximately 5% per cycle, 10% cumulative; ^b ^Approximately 10% per cycle and 17% cumulative;

^c^Approximately 28% per cycle and 42% cumulative; ^d ^Approximately <15%; ^e ^Approximately 50%;

^f^Approximately 4% per cycle, 7% cumulative; ^g ^Approximately 22% per cycle, 32% cumulative;

Date extracted from Gleicher et al [31].

AMH 1.05 ng/mL, thus, represents a distinct point of separation between poorer and better live birth chances: Up to AMH 1.05 ng/mL the chance of live birth per treatment cycle is only approximately 5 percent. Above that, chances are significantly improved [31].

**Figure 5:**
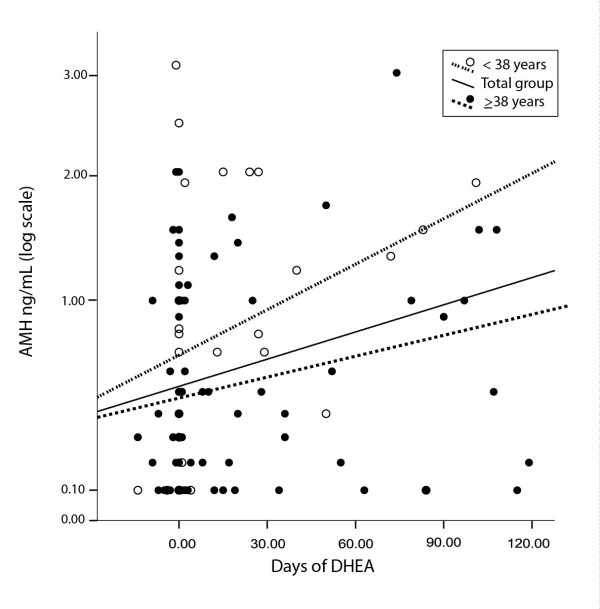
**AMH in POA and DOR patients over time of DHEA exposure**. As the figure demonstrates, AMH increases significantly with length of DHEA treatment (**------**). This effect is more pronounce in young POA patients (**- - -**) than older DOR patients (**......**). Modified with permission from Gleicher et al [32].

AMH increases in parallel to length of DHEA supplementation, and this increase is more pronounced in younger POA than older DOR patients [32] (Figure 5). Moreover, improvements in AMH are statistically highly predictive of pregnancy success [32] but do not yet allow for accurate prediction of who will and will not conceive with DHEA supplementation. AMH responses to DHEA, however, facilitate proper informed consent, particularly important in view of recent ethics guidelines on fertility treatments in poor prognosis patients [33].

#### Treatment protocols, side effects and complications

Except for the previously noted studies by the Baylor group, few other pharma studies have address DHEA utilization, and those were usually restricted to postmenopausal women [34]. Building on the Baylor group's work, the index patient supplemented with micronized DHEA. She utilized over-the-counter products, which have been found inconsistent [35]. Though products are now, likely, improved, we primarily utilize pharmaceutical grade, compounded DHEA, by prescription at a dosage of 25 mg TID. Other authors, including Wiser et al in their recently published clinical trial [12], have used the same dosage of DHEA.

No studies on maximal dosaging of DHEA have, however, been reported, nor have delivery systems been compared. The Baylor group demonstrated distinct advantages from micronized and orally delivered DHEA [6].

Side effects at these dosages are small and rare, and primarily relate to androgen effects.

They include oily skin, acne vulgaris and hair loss. More frequently, patients comment on improved energy levels and better sex drive. In over 1,000 patients supplemented with DHEA, we did not encounter a single complication of clinical significance. A recent paper from Israel reported a posttraumatic seizure after one month of DHEA supplementation in attempts to improve oocytes yields [36]. Except for the anecdotal association, there appears no clinical significance to this report. Even long-term therapy of DHEA, in similar dosages as described here, has been demonstrated safe [37].

DHEA was recently listed amongst drugs with "orphan indications" in fertility therapy [38]. Our center, nevertheless, requires a DHEA-specific informed consent before treatment start.

Two other indications for DHEA supplementation are currently still under investigation in randomized, placebo controlled trials. Those are unexplained infertility (ClinicalTrials.gov ID#NCT00650754) and POF/POI (ClinicalTrials.gov ID# NCT00948857).

#### How does DHEA affect OR?

How DHEA improves OR, IVF parameters, pregnancy chances and decreases miscarriage rates is, ultimately, still unknown. Improved embryo ploidy may, at least in part, explain improvements in miscarriage rates, spontaneous pregnancies and pregnancies after IVF since this would suggest a method of pharmacological embryo selection.

Hodges et al suggested that treatments can be developed which will reduce the risk of age-related aneuploidy by influencing meiotic chromosome segregation [39]. Major disturbances in chromosome alignments on the meiotic spindle of oocytes (congression failure), responsible for aneuploidy, result from the complex interplay of signals regulating folliculogenesis, and increase the risk of non-disjunction errors. Discussed in more detail below, DHEA may, indeed, represent a first such treatment!

Other DHEA effects have, however, also to be considered: The Baylor group suspected increased ovarian IGF-1 to be responsible for observed DHEA effects [1,8]. IGF-1, indeed, appears reduced in poor responders [40].

Androgens, in general, may enhance ovarian function: Already a few decades ago, androgens were in the mouse reported to increase follicle recruitment [41]. Increasing intrafollicular androgens augments granulose cell AMH and inhibin-B production [42].

Androgen receptors have been described in ovarian stroma and granulose cells of primordial follicles, primary follicles and at more advanced stages of folliculogenesis [43]; and ovarian androgens but not estrogens correlate with systemic inflammation during ovarian stimulation with gonadotropins [44].

Frattarelli and Peterson reported that day three testosterone levels below 20 ng/dL are associated with poorer IVF pregnancy rates [45]. They later reported an association with IVF stimulation parameters but not with pregnancy chance [46]. Iranian investigators recently reported that testosterone on day 14 after embryo transfer is predictive of pregnancy chance [47]. Lossl et al published contradictory papers, one claiming [48] and one refuting [49] that treatment with aromatase inhibitors (increasing androgens) improves embryo quality. Contradictory results have also been reported by French investigators in regards to short-term transdermal testosterone administration, with Massin et al reporting no benefit [50], and the Balasch's group in two publications stressing beneficial effects on ovarian resistance [51,52]. The most recent study on the subject by Kim et al demonstrated that transdermal testosterone appears to improve ovarian response to stimulation and IVF outcome in low responders [53].

The, likely, most important study in support of essential androgen effects on follicle development and normal female fertility was recently, however, reported by Sen and Hammes [13]. These two authors were encouraged towards their study by previously reported observations in global androgen receptor knockout (ARKO) female mice, characterized by reduced androgen signaling, and subfertility. The mice also demonstrate defective folliculogenesis, decreased antral follicle counts and corpora lutea, exhibit higher granulose cell apoptosis, are resistant to ovarian stimulation with gonadotropins and often develop POF.

While androgen excess in animal and human experience has widely been associated with excessive and unregulated follicle formation, Sen and Hammes suspected that androgen signaling via androgen receptors may actually be important for normal follicle development and function. Since androgen receptors are widely expressed in different cell types, they decided to determine which androgen receptor - expressing cells contribute to ovarian function and fertility in female ARKO mice.

Using this elegant mouse model they concluded that almost all reproductive phenotypes they observed in global ARKO mice can be explained by lack of androgen receptor expression in granulose cells. Granulosa cell - specific androgen receptors, indeed, appear to promote preantral follicle growth and to prevent follicle atresia. The authors, therefore, concluded that androgen receptors (and by extension androgens) are essential for normal follicle development and female fertility [13].

### Speculating about the future

#### A new concept of age-related declining fecundity

IVF has revolutionized infertility care since it offers tools to maximize pregnancy chances while minimizing multiple pregnancy risks [54]. Even in association with DOR IVF has radically changed the clinical outlook, with pregnancy and live birth rates in women at even advanced reproductive ages constantly improving [31,32,55]. In the US women above age 40, now, represent the most rapidly growing age group giving birth [55].

**Figure 6:**
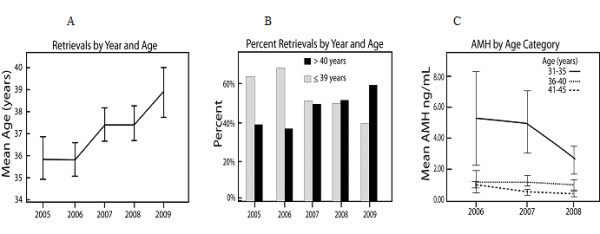
**Trends in patient characteristics of our center's IVF population**. Panel A demonstrates mean ages for IVF patients between 2005 and year-to-date 2009. Panel B demonstrates proportional shift from younger patients (<39 years) to older women (≥ 40 years). Panel C demonstrates that this age shift is also accompanied by a significant fall in AMH levels in younger women (ages 31-35 years) and, therefore, increasing DOR in these younger (POA) patients. Combined, these data explain why in 2009 close to 90% of the center's population was affected by either POA or DOR.

Since young women with normal age-appropriate OR conceive quickly, POA and/or DOR patients, due to their lower pregnancy chances, disproportionally accumulate in infertility centers, needing more IVF cycles. The mean age of newly presenting patients at our center during 2009 was above 39.5 years. Premature or age-dependent DOR represented close to 90 percent of IVF cycle activity (Figure 6). DOR is, therefore, assuming increasing clinical importance, and potentially effective clinical approaches, like DHEA, are attracting wide attention.

Pharmaceuticals, stimulating ovaries, have been at the center of clinical and research interests in reproductive medicine for the last five decades. All agents developed and/or investigated affect follicle maturation, though only during final stages, the so-called gonadotropin-sensitive last two weeks. Here reviewed DHEA effects, in contrast, appear to affect folliculogenesis at much earlier stages.

If confirmed, these observations on DHEA could open ovarian stimulation to radically new horizons, for the first time directing pharmacological interventions towards earlier stages of in vivo follicle maturation.

Declining female fecundity with advancing age is based on diminishing follicle numbers and deteriorating egg quality [14,15,56]. While declines in follicle numbers are undisputed, the here presented DHEA experience raises, however, questions about the widely held understanding that declining egg quality with advancing female age is caused by aging oocytes.

Young women with prematurely DOR exhibit most typical signs of ovarian aging, such as elevated FSH, low AMH and ovarian resistance to stimulation, but do not demonstrate increased aneuploidy [57]. Their oocytes thus, quite obviously, are functionally not behaving "old" enough to lead to aneuploidy, while oocytes from older women, indisputably, demonstrate increased aneuploidy [27,29].

We previously noted that DHEA supplementation apparently significantly reduces these age-related increases in aneuploidy [25,26], and, therefore, also reduces age-associated increases in miscarriages [28,31]. In absence of healthy and genetically normal oocytes both of these findings are inconceivable. DHEA is, therefore, either able to revert "older," already damaged oocytes, into "younger" oocytes, in itself a rather unlikely proposition, or one has to conclude that, contrary to current dogma [15,56], oocytes in their resting stages within unrecruited primordial follicles do not really age.

Once recruited, they, however, enter age-dependent ovarian environments where follicle maturation takes place. These ovarian environments can be of different quality and will, uniformly, deteriorate as women age. As proposed by Hodges et al, these environments affect segregation processes during meiosis, giving rise to increased aneuploidy at older ages. Hodges and associates, however, also pointed out that these envrironments, and with it aneuploidy and miscarriage rates, may be open to pharmacologic manipulation [39].

Here presented DHEA data, therefore, support the concept that ovarian environments, but not resting oocytes, age as women grow older. Under such a concept here described DHEA effects are perfectly understandable. DHEA levels, indeed, peak in humans between ages 20 and 30 years, and then decline by approximately 2 percent per year, to reach nadirs of 10 to 20 percent around age 80 years [58].

In some women, aneuploidy may, thus, simply, represent a reversible DHEA deficiency. Others may lack yet to be determined components of healthy ovarian environments and, therefore, may benefit from other supplementations.

Proven correct, one can expect a significant expansion of the female's reproductive lifespan as science learns how to reconstitute ovarian environments, mimicking conditions of younger ages. Since even menopausal ovaries still contain follicles and oocytes [14], at least theoretically, childbirth may be expandable into the 50s.

In the Squirrel monkey, older animals, immediately prior to cessation of reproduction, still demonstrate an abundance of well-differentiated granulosa cells [58]. Assuming that unrecruited oocytes maintain their youth and that aged ovarian environments can be rejuvenated, smaller, but healthier, egg cohorts may, indeed, allow for pregnancy into surprisingly advanced female ages.

DHEA may, therefore, represent a first compound in a new category of pharamacological agents with potential to "rejuvenate" ovarian environments. Following a similar concept, Bentov et al., based on the known loss of mitochondrial functions with advancing age, recently suggested the use of mitochondrial nutrients, like coenzyme Q10 (CoQ10), after demonstrating that CoQ10 increases oocytes numbers in older mice [59]. Androgens positively affect mitochondrial function [60].

A better understanding of differences in ovarian environment between younger and older women will be needed to discover additional beneficial pharmacological agents. The technology for such studies is being developed [61].

### Utilization of DHEA outside of infertility

Ovarian aging does not only affect infertile women. Age-dependency of fecundity is driven by the acknowledgment that ovaries age with expected adverse consequences, including longer times to conception, increased aneuploidy and increased spontaneous miscarriages risks.

One, therefore, can also conceive of potentially utilizing DHEA, and other pharmaceuticals able to rejuvenate ovarian environments, in normally fertile, older women attempting to conceive. Like supplementation with folic acid to prevent neural tube defects [62], supplementation with DHEA may achieve favorable public health consequences by potentially reducing aneuploidy and spontaneous pregnancy losses in a general population.

### Limitations

Despite worldwide utilization of DHEA supplementation in women with DOR, lack of enough controlled studies is still regretful. With the small study by Wiser et al [12] representing the only prospective clinical trial (Level I evidence), studies of more substantial size are all based on lower levels of evidence and, therefore, have to be interpreted cautiously. This fact is reemphasized by most publications coming from only a small number of centers, including, these authors' own center.

Purists may argue that no treatments should be routinely applied in clinical practice, unless based on prospectively randomized studies. Recognizing that Level I clinical trials may, at times, be too costly and/or too difficult to conduct, such an approach has, however, recently been questioned in the academic community [63-65].

## Conclusions

Best available evidence for the utilization of DHEA supplementation in improving ovarian performance in women with DOR was reviewed. A small, recently published clinical trial [12] and remarkable animal data [13] offer increasingly convincing clinical and experimental support for the use of DHEA, and possibly other androgens, in women with DOR.

These newly available data add to Level II and III evidence, generated by a small number of investigators, these authors included, over the preceding six years. Combined, these data suggest that DHEA supplementation may be effective in improving pregnancy chances in women with DOR. Since a DOR diagnosis often leaves limited time for treatment, patients should be given the choice of DHEA supplementation, though with appropriate informed consents. Especially with severe DOR, DHEA may, at least in some patients, make the difference between conceptions with autologous or heterologous oocytes.

Considering absence of significant side effects and, at least within the US, availability of DHEA as a food supplement, here presented data support utilization of DHEA in association with DOR, though attempts should be made to further define best suited patient populations for such treatment, maximally effective treatment protocols and best delivery systems.

## Abbreviations

AMH: Anti-Müllerian hormone; ARKO: Androgen receptor knock out; CoQ10: Coenzyme Q10; DHEA: dehydroepiandrosterone; DOR: diminished ovarian reserve; IVF: In vitro fertilization; OPOI: Occult primary ovarian insufficiency; OR: Ovarian reserve; PGS: Preimplantation genetic screening; POF: Premature ovarian failure; POI: Primary ovarian insufficiency; U.S.: United States;

## Competing interests

Unless otherwise noted, each author and spouse/life partner (if any) has nothing to disclose. Both authors are listed as co-inventors of a U.S. patent, which claims beneficial effects from DHEA supplementation in women with diminished ovarian reserve on ovarian function and pregnancy rates. Both authors are also listed as inventors on other, still pending patent applications in regards to DHEA effects on ovarian function, and on other patents, unrelated to the topic of this communication. Neither author derives financial benefits from any of these patents. Both authors received in the past research support, speaker honoraria and travel funds from various pharmaceutical and medical device companies, though none of these companies is related in any fashion to the topic, covered in this manuscript.

## Authors' contributions

NG and DHB contributed equally to this manuscript. Both authors read and approved the final manuscript.
